# Type IIB DNA topoisomerase is downregulated by trastuzumab and doxorubicin to synergize cardiotoxicity

**DOI:** 10.18632/oncotarget.23543

**Published:** 2017-12-21

**Authors:** Jiangsong Jiang, Nishant Mohan, Yukinori Endo, Yi Shen, Wen Jin Wu

**Affiliations:** ^1^ Division of Biotechnology Research and Review I, Office of Biotechnology Products, Office of Pharmaceutical Quality, Center for Drug Evaluation and Research, US Food and Drug Administration, Silver Spring, MD, USA

**Keywords:** trastuzumab, DNA topoisomerase 2B (TOP2B), doxorubicin, cardiotoxicity, HER2

## Abstract

Despite heightened risk of cardiotoxicity associated with combination therapy of anthracyclines and trastuzumab in HER2-positive breast cancer patients, little research effort has been invested in exploring the molecular mechanisms of cardiotoxicity induced by this combination therapy. In this study, we demonstrate that trastuzumab downregulates both gene and protein expressions of type IIB DNA topoisomerase/DNA topoisomerase IIB (TOP2B), a major intracellular target mediating doxorubicin-induced cardiotoxicity, in human primary cardiomyocytes. This in turn induces DNA damage activity and DNA double strand breaks, which is indicated by the enhanced phosphorylation of H2AX (γH2AX) and ataxia telangiectasia and Rad3-related protein (ATR pS428) in trastuzumab-treated cardiomyocytes. Furthermore, concurrent or sequential treatment of doxorubicin and trastuzumab significantly increases the downregulation of the protein levels of TOP2B, enhances apoptosis and cell growth inhibition, and promotes production of reactive oxidative and nitrative species in human cardiomyocytes as compared to either trastuzumab or doxorubicin treatment, indicating augmentation of cardiotoxicity in combination therapy. Additionally, our data reveal that doxorubicin treatment increases the levels of ErbB2/HER2 expression in human cardiomyocytes as compared with that in cells not treated with doxorubicin, leading to the enhanced activity downstream of HER2 signaling. Consequently, this may render the cardiomyocytes to become addicted to HER2 signaling for survival under stressed conditions. Enhanced HER2 protein expression leaves cardiomyocytes more sensitive to trastuzumab treatment after doxorubicin exposure. This study provides molecular basis for significantly increased cardiotoxicity in cancer patients who are treated with anthracyclines and trastuzumab-based combination regimens.

## INTRODUCTION

Chemotherapy followed by trastuzumab in adjuvant setting is the standard of care for the treatment of patients with ErbB2/HER2-positive breast cancers [1–3]. Clinical regimens consisting of doxorubicin and trastuzumab have shown significant improvement in cancer patient mortality and morbidity, however, cardiac dysfunction and heart failures associated with combination regimens continue to pose challenges to clinicians. In an early clinical trial encompassing 469 patients to evaluate safety and efficacy of trastuzumab, cardiac dysfunction was reported to occur in up to 27% in anthracycline, cyclophosphamide, and trastuzumab group; 8% in anthracycline and cyclophosphamide group; 13% in paclitaxel and trastuzumab group; and 1% in paclitaxel alone group [4]. Likewise, a retrospective study, which evaluated the incidence of cardiac abnormalities in HER2-positive cancer patients receiving adjuvant trastuzumab concluded that approximately 1 in 4 women might develop left ventricular systolic dysfunctions after treatment with trastuzumab in adjuvant settings [5]. Despite the improved patient inclusion and exclusion criteria, concurrent and sequential therapy regimens consisting of anthracyclines and trastuzumab continue to cause significant cardiotoxicity in succeeding clinical studies. However, it should be noted that cardiotoxic events from recent clinical studies are not as high as early clinical trials as reported previously [6, 7]. A recent clinical study with 18,540 young adult women showed that the estimated cumulative incidence of major cardiac events was 6.6% for the anthracycline-trastuzumab sequential therapy, 5.1% for trastuzumab without anthracyclines, 2.0% for anthracyclines without trastuzumab, and 3.2% for other chemotherapy over 3 years [7]. This study concluded that patients receiving sequential therapy have higher risk of developing hospital-based cardiac dysfunctions compare with patients receiving anthracycline alone [7].

The DNA topoisomerase II (TOP2) is an enzyme that catalyzes the transient breaking and adjoining of DNA double helix and plays critical role in nuclear processes such as chromosome condensation, chromatid separation and supercoiling relaxation during DNA transcription and replication [8, 9]. The tumoricidal properties of doxorubicin are attributed to its ability to form ternary complex with homodimeric TOP2 subunits and DNA, causing DNA double strand breaks and DNA damage in tumor cells [10, 11]. In cardiac cells where DNA topoisomerase IIB (TOP2B) is highly expressed, doxorubicin conjugates with TOP2B protein and DNA and induces double strand breaks of DNA, activating DNA damage response and apoptosis [12, 13]. It has been hypothesized that doxorubicin-mediated DNA damaging response and apoptosis instigates remarkable alteration in transcriptome that specifically targets genes responsible for oxidative phosphorylation and mitochondrial biogenesis in cardiomyocytes leading to cardiac cell death [13]. In addition to direct DNA damage, the low concentrations of doxorubicin can increase its oxidative metabolism in which quinone structure of doxorubicin is oxidized to semiquinone free radicals in presence of NADPH enzymes and generate massive reactive oxygen species (ROS) leading to cardiac cell death [14, 15]. Doxorubicin treatment triggers excessive accumulation of iron inside the mitochondria, which increases cellular ROS levels and induces cardiomyocyte damage through mitochondrial dysfunction [15, 16]. Rac1, an integral subunit of NADPH oxidase, contributes towards doxorubicin-mediated cardiomyocyte apoptosis and oxidative stress in ROS-dependent and ROS-independent manner [17]. Rac1-specific inhibitor NSC23766 (NSC) has been shown to exhibit protective effects in mitigating doxorubicin-induced cardiotoxicity by reducing doxorubicin-induced DNA damage marker levels and repairing DNA damage response [18]

Trastuzumab is a humanized monoclonal antibody directed against extracellular domain IV of human epidermal growth factor receptor 2 (HER2) and approved for treatment of HER2-positive breast and gastric cancers [19, 20]. Despite tremendous efforts to understand the trastuzumab-mediated cardiac dysregulations, cellular and molecular aspects of cardiotoxicity remain elusive. Our previous investigation revealed that trastuzumab prominently alters the expression of genes essential for cardiac and mitochondrial functions, DNA repair and oxidative damage and induces ultrastructural modifications in C57BL/6 mice model [21]. HER2 knockout models have indicated the critical role of HER2 signaling in cardiac development and global cardiac functions [22]. Recent investigation from our lab demonstrated that trastuzumab impairs HER2 signaling and inhibits autophagic machinery to augment ROS production in the primary human cardiomyocyte [23]. In clinical settings, doxorubicin causes cardiotoxicity in dose dependent manner and cardiomyocyte damage begins immediately after administration of the first dose [24]. As cumulative exposure increases, the permanent and irreversible injury to myocyte occur proportionately which is categorized as type I cardiotoxicity in clinical settings [24]. It is still a matter of debate as to whether or not trastuzumab-induced cardiotoxicity is reversible [25, 26].

In the present study, we identified that TOP2B, which is widely implicated in doxorubicin-induced cardiotoxicity, is down-regulated by trastuzumab treatment in human cardiomyocyte model. We examined whether trastuzumab-mediated TOP2B down-regulation can enhance DNA damage and DNA double strand breaks in human cardiomyocytes that are exposed to trastuzumab as single agent. In order to mimic trastuzumab adjuvant systemic therapy, we treated cardiomyocytes with trastuzumab and doxorubicin either concurrently or sequentially and investigated the molecular basis of cardiotoxicity by monitoring apoptosis, cell growth, and oxidative stress.

## RESULTS

### Trastuzumab, trastuzumab plus pertuzumab and T-DM1 treatments cause gene expression changes in primary human cardiomyocyte

**Figure 1:**
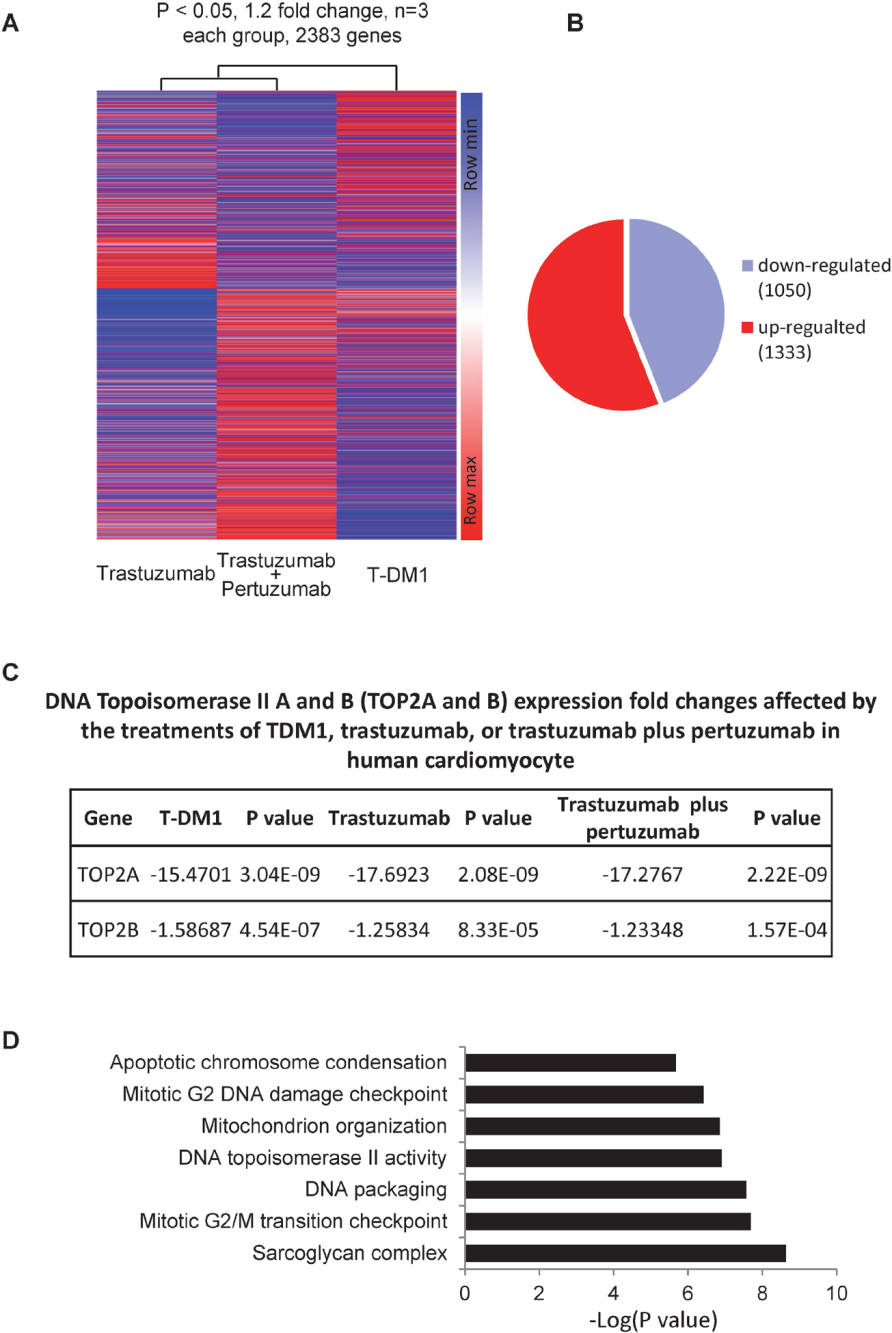
Gene expression changes in human primary cardiomyocyte treated with trastuzumab, trastuzumab plus pertuzumab, or T-DM1 (**A**) Heat map for the changes of gene expression in human primary cardiomyocytes treated with trastuzumab, trastuzumab plus pertuzumab or T-DM1. Data with opposite influence on gene expression among these three treatments were removed, and then were further selected with threshold +/– 1.2 fold changes and *P* < 0.05 as selection criteria. This results in 2383 genes, which were input into the website Morpheus to generate a heat map according to the instruction. Following parameters were used to generate the heatmap with hierarchical clustering (metric: euclidean distance; linkage method: average). (**B**) Number of genes either up- or down- regulated by trastuzumab, trastuzumab plus pertuzumab or T-DM1. (**C**) Gene expression changes in DNA topoisomerase IIA (TOP2A) and IIB (TOP2B) in human primary cardiomyocytes treated by T-DM1, trastuzumab, or trastuzumab plus pertuzumab. (**D**) Gene clusters affected by trastuzumab treatment in human primary cardiomyocyte.

Using a mouse model, we previously showed that trastuzumab alters the expressions of myocardial genes that are essential for normal functioning of cardiac physiology [21]. In this study, we employed DNA microarray platform to compare the gene expression patterns in primary human cardiomyocytes treated with trastuzumab (50 µg/ml), trastuzumab (50 µg/ml) plus pertuzumab (50 µg/ml), T-DM1 (10 µg/ml), or control (no treatment). After data acquisition, statistical methods were applied to evaluate and filter the data based on the statistical significance. We identified 2383 genes with at least 1.2 folds changes in expression level between control and treated samples (Figure 1A). Among those genes, 1050 genes were downregulated by trastuzumab, trastuzumab plus pertuzumab, and T-DM1, whereas 1333 genes were upregulated by trastuzumab, trastuzumab plus pertuzumab, and T-DM1 (Figure 1B). As shown in Figure 1C, both TOP2A & B were downregulated by trastuzumab, trastuzumab plus pertuzumab, and T-DM1. GO ANOVA analysis revealed that several molecular pathways critical for cardiomyocyte’s functions were affected by trastuzumab (Figure 1D).

### Trastuzumab downregulates TOP2A and B protein expression in human cardiomyocytes

**Figure 2:**
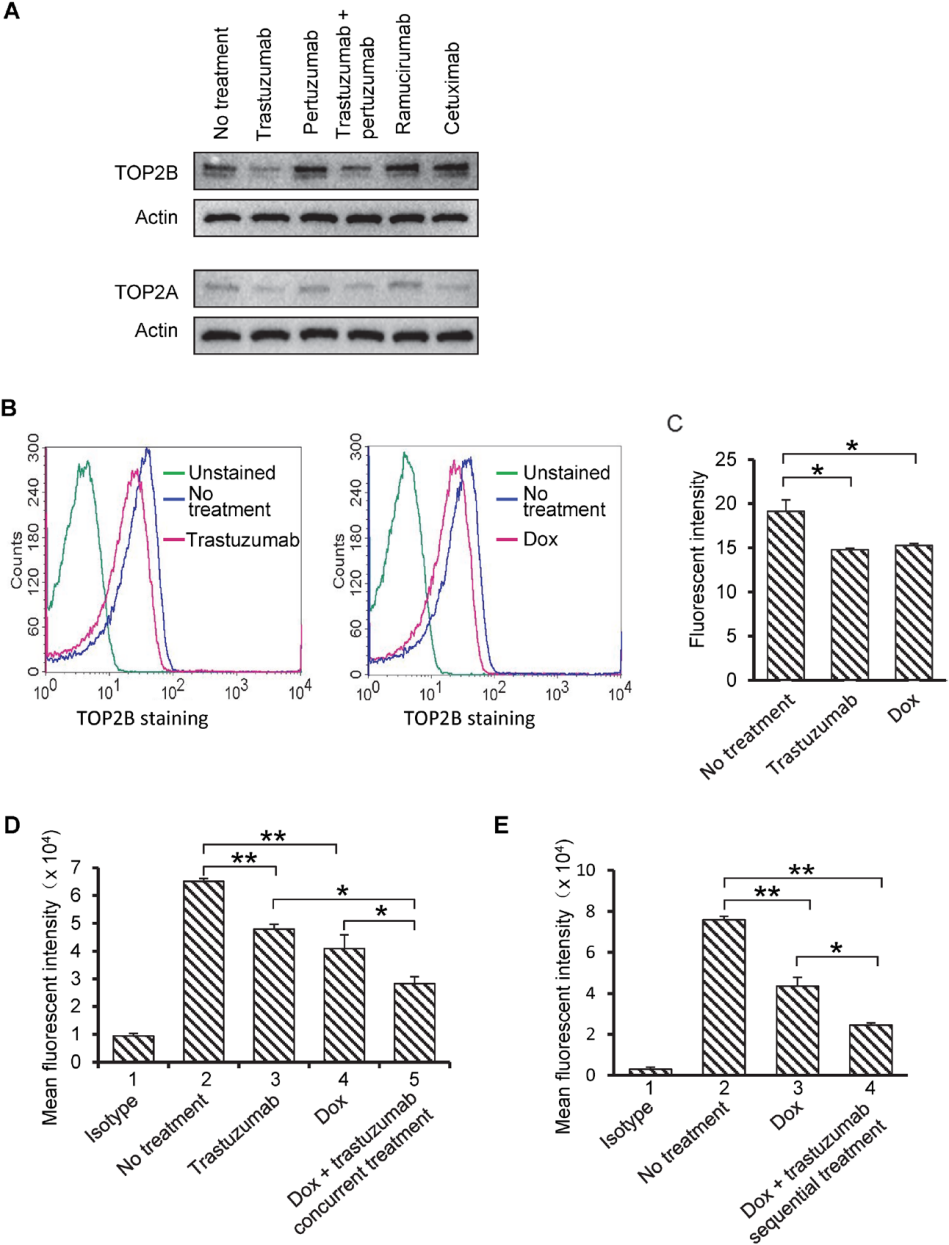
Protein levels of TOP2B are downregulated by either trastuzumab or combination of doxorubicin (Dox) and trastuzumab in primary human cardiomyocytes (**A**) Human primary cardiomyocytes were treated with trastuzumab, pertuzumab, trastuzumab plus pertuzumab, ramucirumab and cetuximab at 50 µg/ml for 24 h. After treatments, Western blotting was performed, and expression levels of TOP2B and TOP2A were detected using antibodies directed against TOP2B or TOP2A. Equal loading of protein samples were confirmed by actin blots. Upper two panels: TOP2B expression; lower two panels: TOP2A expression. (**B**) TOP2B protein levels in human primary cardiomyocytes that were either treated with trastuzumab at 50 µg/ml (left graph) or doxorubicin (Dox) at 0.5 µM (right graph) or left untreated for 24 h. TOP2B expression was monitored using flow cytometry. (**C**) Quantitative analysis of fluorescent intensity obtained from flow cytometry analysis was presented in the form of bar graphs. Data are representative of two or more independent experiments and expressed as mean ± SEM. (**D**) TOP2B protein levels in human primary cardiomyocytes that were treated with trastuzumab (50 µg/ml), Dox (0.5 µM), trastuzumab (50 µg/ml) plus Dox (0.5 µM) or left untreated for 24h. TOP2B protein levels were monitored using flow cytometry. (**E**) TOP2B protein levels in human primary cardiomyocytes that were treated with Dox (0.5 µM) for 24 h or left untreated for 48 h. After 24h Dox treatment, Dox was removed from cell culture media, and cells were then either treated with trastuzumab (50 µg/ml) or left untreated for additional 24h. TOP2B protein levels were monitored using flow cytometry. The isotype in Figure 2D and 2E is a negative control. Experiments were performed in triplicates, and data are representative of two - three independent experiments. Student’s *t*-test was used for statistical significance. ^*^P < 0.05; ^**^P < 0.01.

Cardiac-specific TOP2B deficiency rescued cardiomyocytes from doxorubicin-induced DNA double strand breaks, defective mitochondrial functions and increased ROS generation indicating that TOP2B is a critical mediator of doxorubicin-induced cardiomyopathy [16]. Given that combination of doxorubicin with trastuzumab significantly increases cardiotoxicity in patients and TOP2B is a critical intracellular target of doxorubicin to mediate cardiotoxicity, we investigated the levels of TOP2A and TOP2B protein in cardiomyocytes treated with different antibodies. Human primary cardiomyocytes were treated with trastuzumab, pertuzumab, trastuzumab plus pertuzumab, ramucirumab or cetuximab at 50 µg/ml for 24h, and the protein levels of TOP2A and TOP2B were detected in cell lysates. As shown in Figure 2A, TOP2B protein levels were remarkably decreased in trastuzumab or trastuzumab plus pertuzumab-treated human cardiomyocytes as compared with that in non-treated cells (upper panel). Individual treatment of pertuzumab, ramucirumab or cetuximab did not affect TOP2B expression in human cardiomyocytes (Figure 2A). Previous studies have indicated that TOP2A is expressed relatively low in cardiomyocytes [27]. In our study, we observed that even though TOP2A levels were low in human cardiomyocytes, trastuzumab or trastuzumab plus pertuzumab, but not pertuzumab alone or ramucirumab, decreased TOP2A expression after 24h treatment (Figure 2A, lower panel). TOP2A expression was slightly reduced in cetuximab-treated cells (Figure 2A, lower panel). To further confirm the results shown in Figure 2A, the levels of TOP2B were monitored using flow cytometry. As shown in Figure 2B and 2C, TOP2B protein levels were significantly reduced in human cardiomyocytes exposed to either trastuzumab (50 µg/ml) or doxorubicin (0.5 µM) as compared with that in non-treated cells. Next, we asked the question of whether combination of doxorubicin and trastuzumab can further downregulate TOP2B. As shown in Figure 2D, the levels of TOP2B were significantly reduced in cells treated with doxorubicin plus trastuzumab as compared to that treated with either doxorubicin or trastuzumab. Figure 2E showed that the levels of TOP2B were also significantly reduced in cells treated sequentially with doxorubicin then trastuzumab as compared to that treated with doxorubicin alone.

### Trastuzumab induces DNA lesion response in the primary human cardiomyocytes

**Figure 3:**
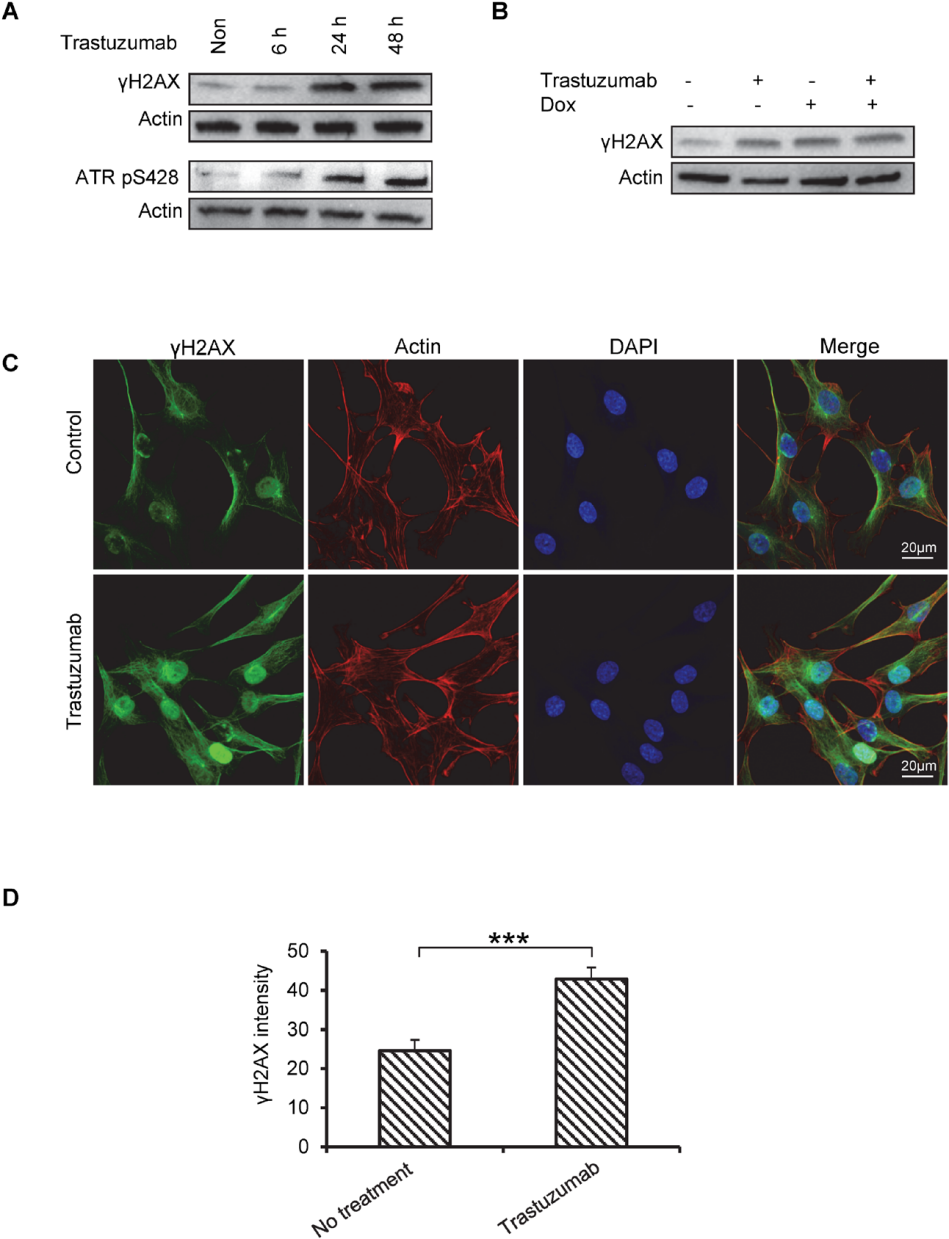
Trastuzumab induces phosphorylation of H2AX and ATR in human primary cardiomyocyte (**A**) Human primary cardiomyocytes were treated with trastuzumab (50 µg/ml). Cells were then harvested at the indicated times, and whole cell lysates (WCL) were loaded on SDS PAGE gel to exam the phosphorylation levels of H2AX (γH2AX) and ATR (ATR pS428) using corresponding antibodies. Actin was used as loading control. (**B**) Human primary cardiomyocytes were treated with trastuzumab (50 µg/ml), dox (0.5 µM), trastuzumab plus Dox or left untreated for 24h. Whole cell lysate was subjected to western blotting, and phosphorylation of H2AX was detected by using anti- γH2AX antibody (**C**) Immunofluorescent images of γH2AX (green) in human primary cardiomyocytes treated with trastuzumab at 50 µg/ml for 24h or left untreated. Actin was co-stained with phalloidin (red) to indicate the cell structure. Cell nuclei were stained with DAPI. (**D**) Quantitative analysis of γH2AX signal in the primary human cardiomyocytes. Statistical significance was determined by the Student’s *t*-test; ^***^P < 0.001.

The histone variant H2AX constitutes about 2.5–25% of total H2A in the mammalian genome and plays a key role in repair process of damaged DNA. Histone H2AX is phosphorylated by kinases such as ataxia telangiectasia mutated (ATM) and ataxia telangiectasia and Rad3-related protein (ATR) during DNA damage response [28]. The phosphorylated protein, γH2AX, initiates the recruitment and localization of DNA repair proteins, therefore, is frequently used as a biomarker for DNA damage and DNA damage response (DDR) [28, 29]. We monitored the effect of trastuzumab at S139 phosphorylation of histone H2AX in human cardiomyocytes to assess DDR triggered by DNA double-strand breaks (DSBs). As shown in Figure 3A, the levels of γH2AX protein began to increase at 6h and peaked at 24h and 48h of exposure to trastuzumab (Figure 3A, upper panel). H2AX can also be phosphorylated by ataxia telangiectasia and Rad3-related protein (ATR), and the phosphorylation of ATR at Ser428 (ATRpS428) serves as an important marker for DNA lesion [28]. Figure 3A showed that ATRpS428 levels were increased after 24 h treatment with trastuzumab. Additionally, phosphorylation of H2AX in cardiomyocytes was also enhanced in the cell treated with either Dox or Dox plus trastuzumab coucurrently for 24 h (Figure 3B). Further confirmation with fluorescent immunostaining using confocal microscopy concluded that there was a significant increase in γH2AX levels in the nuclei of trastuzumab-treated cardiomyocytes as compared to control cells (Figures 3C and 3D).

### Trastuzumab potentiates the effect of doxorubicin on programed cell death in human cardiomyocytes

**Figure 4:**
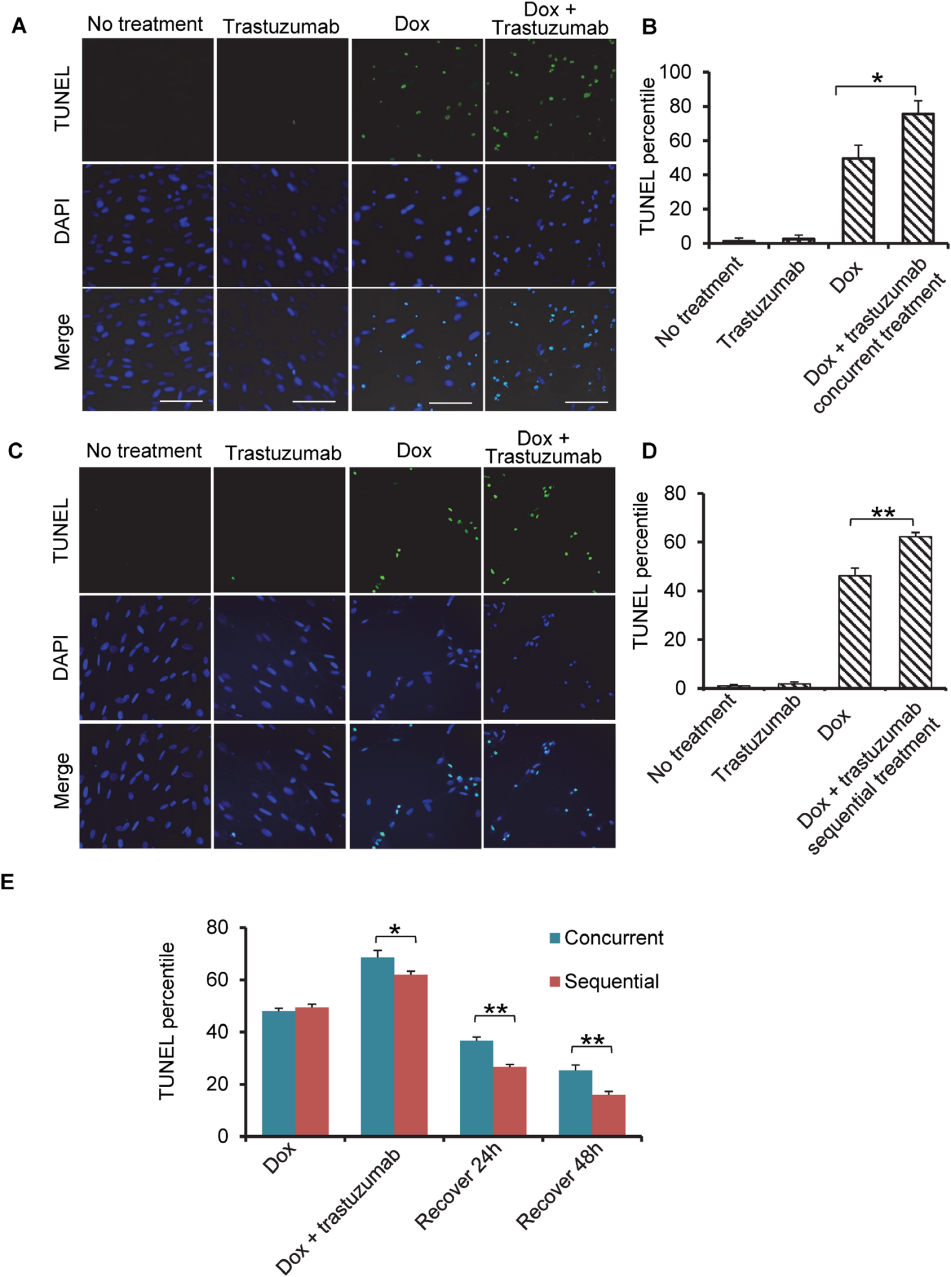
Trastuzumab enhances Dox-induced apoptosis synergistically in human primary cardiomyocyte (**A**) Cells were treated with trastuzumab (50 µg/ml), Dox (0.5 µM) or the concurrent combination of these two drugs for 24 h, and TUNEL assay followed by fluorescent confocal microscopy were carried out to obtain the images. (**B**) Quantitation of cells undergoing the apoptosis in the experiment described in Figure 4A. (**C**) Sequential treatment of cardiocytes with Dox (0.5 µM) for 24 h followed by trastuzumab (50 µg/ml) for additional 24 h. TUNEL assay performed was the same as that described in Figure 4A. (**D**) Quantitation of cells undergoing the apoptosis in the experiment described in (C). (**E**) Quantitation of TUNEL assay at different time points after doxorubicin and trastuzumab were withdrawn from either concurrent or sequential treatment as described in Figure 4A and 4C. Cells were grown on coverslip, and after treatment with doxorubicin and trastuzumab in either concurrent or sequential , cells were left to recover in cell culture media without any drugs. Coverslips were fixed for staining immediately after drugs withdrawn or after 24 h or 48 h recovery. Doxorubicin treatment was used as control. Statistical significance was determined by the Student’s *t* test ; ^*^P < 0.05.

Anthracyclines combined with trastuzumab is used for the treatment of patients with HER2-positive breast cancers. Using TUNEL assay, we studied the programmed cell death in the human cardiomyocytes treated with trastuzumab, doxorubicin or trastuzumab combined with doxorubicin in concurrent settings for 24h. Trastuzumab treatment (50 µg/ml) of cardiomyocyte did not cause any significant cell death, while 0.5 µM doxorubicin treatment induced a dramatic cell death (53%) (Figure 4A and 4B). Moreover, doxorubicin plus trastuzumab significantly increased the rate of programmed cell death to 72% (Figure 4A and 4B). TUNEL assay also showed significant programmed cell death in human cardiomyocytes after doxorubicin and trastuzumab treatment in sequential settings (Figure 4C and 4D). These data demonstrated that trastuzumab was able to significantly increase programmed cell death following doxorubicin treatment in both concurrent and sequential treatment settings.

Next, we ascertained whether human cardiomyocytes can recover from programmed cell death induced by doxorubicin and trastuzumab combination treatments. To model this, human cardiomyocytes were exposed to doxorubicin and trastuzumab in either concurrent or sequential manner as indicated in Figure 4A and 4C, and then allowed to recover for 24h and 48h. While statistical analysis from recovery experiments indicate that the percentage of programed cell death was reduced in both concurrent and sequential treatments and 48 h recovery was more prominent than 24h, cardiomyocytes recovered more significantly from cell death in sequential treatment group than concurrent treatment group (Figure 4E).

### Trastuzumab enhances caspase 3/7 activation and inhibits cardiomyocyte growth when applied with doxorubicin sequentially

**Figure 5:**
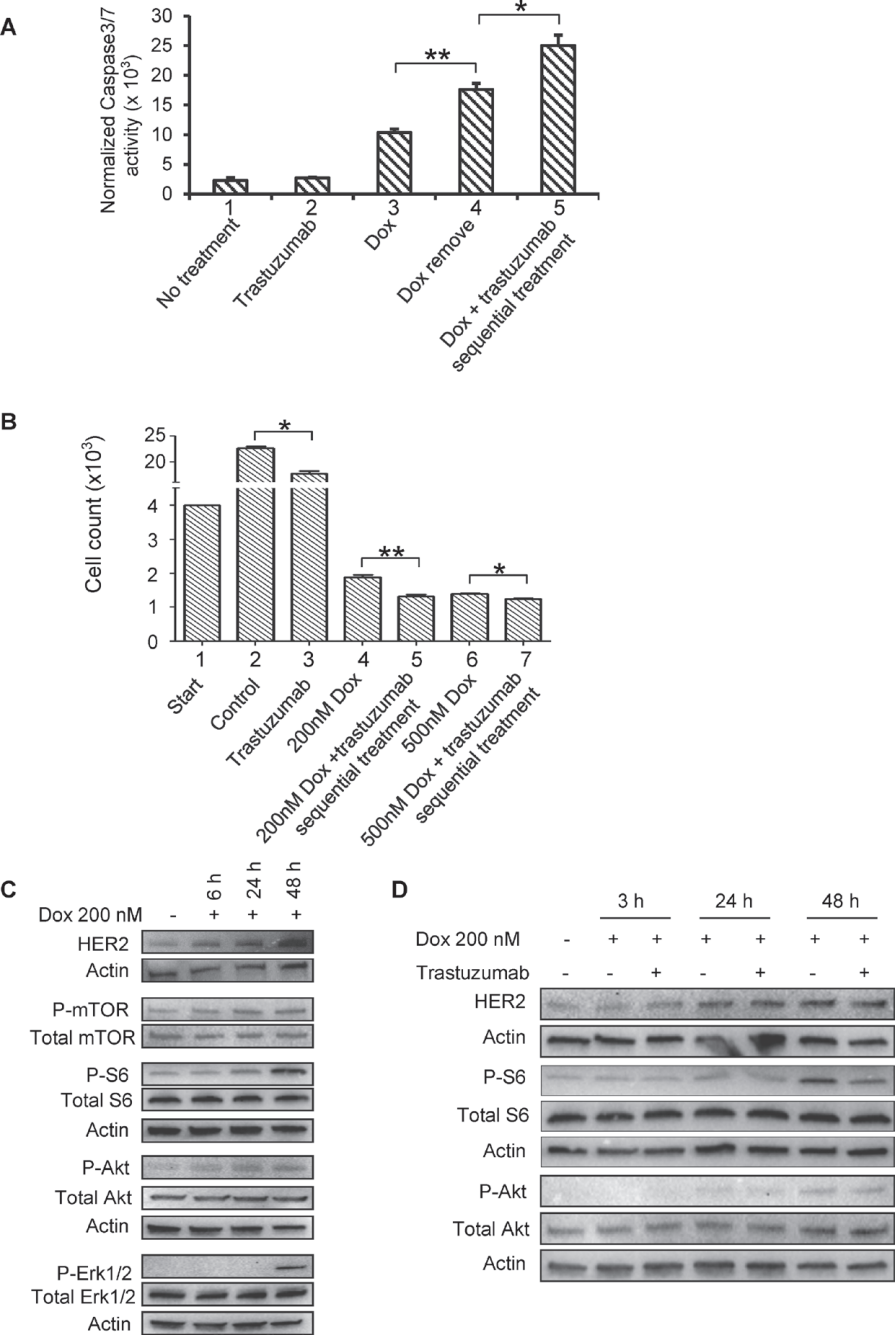
Sequential treatment of Dox followed by trastuzumab enhances caspase 3/7 activity, and Dox has the ability to modulate HER2 signaling (**A**) Caspase 3/7 activity was measured in human cardiomyocytes left untreated or treated with trastuzumab (50 µg/ml), Dox (0.5 µM) or Dox followed by trastuzumab. Data are representative of three independent experiments and expressed as mean ± SEM. Statistical significance was determined by the Student’s *t*-test; ^*^, *P* < 0.05; ^**^, *P* < 0.01. (**B**) Cell growth after treatment with control, trastuzumab (50 µg/ml), Dox (0.2 µM and 0.5 µM) or Dox (0.2 µM and 0.5 µM) followed by trastuzumab (50 µg/ml). Data are representative of three independent experiments and expressed as mean ± SEM. Statistical significance was determined by the Student’s *t*-test; ^*^P < 0.05; ^***^P < 0.001. (**C**) Dox induces HER2 expression in human primary cardiomyocytes in a time-dependent manner. Human primary cardiomyocytes were seeded in a 6-well plate and treated with Dox at 200 nM for the indicated times. The expression levels of HER2, and phosphorylated mTOR (P-mTOR), S6 (P-S6), Akt (P-Akt), and Erk1/2 (P-Erk1/2) were determined by Western blotting analysis. (**D**) Human primary cardiomyocytes were treated with either Dox (0.2 µM) alone or in combination with trastuzumab (50 µg/ml) for the indicated times. Cells were harvested and WCL were subjected to Western blotting analysis to detect the expression levels of HER2 and phosphorylated S6, Akt and Erk1/2.

In order to further understand the cardiotoxicity induced by the combination of doxorubicin and trastuzumab, we examined the caspase 3/7 activation after the cardiomyocytes were treated with doxorubicin, trastuzumab, or both of them in a sequential manner. Our data showed that trastuzumab alone did not significantly activate caspase 3/7 (Figure 5A, column 2), which is consistent with the programmed cell death event observed with TUNEL assay (Figure 4). Accordingly, doxorubicin can induce caspase 3/7 activation after 24h treatment (Figure 5A, column 3). Even though doxorubicin was removed from the medium after treatment, and cells were left to recover for another 48h, caspase 3/7 activity continued to increase (Figure 5A, column 4). Furthermore, caspase 3/7 activity was further increased in cells that were pre-treated with doxorubicin for 24h and then trastuzumab for additional 24 h (Figure 5A, compare column 4 with column 5). These results showed that sequential treatment of doxorubicin followed by trastuzumab added more toxicity to cardiomyocytes.

Next, we evaluated the cell growth inhibition of trastuzumab either alone or sequential application of doxorubicin followed by trastuzumab. 4 × 10^3^ cells were seeded in 96-well plates overnight and then treated or left untreated with trastuzumab for 48h and counted (Figure 5B, columns 2 and 3). In the same experiment, cells were also treated with doxorubicin with two indicated doses for 24h, and doxorubicin then was removed from the cell culture media. Next, the doxorubicin pre-treated cells were either treated with trastuzumab (Figure 5B, columns 5 and 7) or left untreated (Figure 5B columns 4 and 6) for additional 24h counted. As shown in Figure 5B, trastuzumab alone had minor effect on cell number. Notably, doxorubicin can significantly reduce cell number at 200 nM or 500 nM dose levels. When trastuzumab was applied to the doxorubicin-pre-treated cells, cell number was further reduced (Figure 5B). Taken together, these data suggest that sequential treatment of doxorubicin followed by trastuzumab exhibits additive inhibition on cardiomyocyte growth.

### Doxorubicin increases the levels of HER2 expression and modulates downstream of HER2 pathway

Doxorubicin treatment has also been shown to increase phosphorylation of the ribosomal protein S6 in mice model, which is a downstream substrate of mTOR/S6K implicated in cardiomyocyte growth [30]. Since mTOR is the downstream of HER2 signaling, we asked question of whether doxorubicin is able to influence HER2 signaling in cardiomyocytes. As shown in Figure 5C, the levels of HER2 expression in human cardiomyocytes were increased after the treatment of cardiomyocytes with doxorubicin in a time-dependent manner (Figure 5C). Consistent with previously reported [30], we also found that doxorubicin-mediated phosphorylation of S6 was increased in human cardiomyocytes in a time-dependent manner, while doxorubicin treatment modestly enhanced the levels of mTOR phosphorylation (Figure 5C). The levels of phosphorylated Akt were slightly increased. Interestingly, we observed a delayed, but strong response in Erk phosphorylation in doxorubicin-exposed cardiomyocytes (Figure 5C), which was probably the consequence of upregulated HER2 expression.

In order to study whether combination therapy of doxorubicin and trastuzumab can downregulate doxorubicin-induced upregulation of HER2 expression and mTOR signaling activation, we treated cardiomyocytes with either doxorubicin or doxorubicin plus trastuzumab for 3h, 24h and 48h. We further confirmed that HER2 expression was increased by doxorubicin in cardiomyocytes in a time-dependent manner (Figure 5D). However, the Western blotting data indicated that p-S6 level, which was considerably increased in doxorubicin-treated cells as compared with non-treated cells, was greatly downregulated by trastuzumab in doxorubicin-treated cells (Figure 5D). A slight decrease in Akt activity was also found in the cells treated with doxorubicin followed by trastuzumab as compared with that in doxorubicin only-treated cells, whereas Erk activity was not affected by doxorubicin followed by trastuzumab (data not shown). Taken together, we proposed a novel molecular mechanism by which combination of doxorubicin and trastuzumab increases cardiotoxicity. Specifically, doxorubicin treatment upregulates HER2 signaling, which is likely a compensatory or cell survival response to doxorubicin treatment. On the other hand, the upregulated-HER2 signaling may render the cells to become addictive to HER2-mediated signaling. This may leave cardiomyocytes to be more vulnerable or sensitive to trastuzumab treatment as compared with cardiomyocytes that are not exposed to doxorubicin treatment.

### Sequential treatment of doxorubicin followed by trastuzumab increases ROS and RNS production in human cardiomyocytes

**Figure 6:**
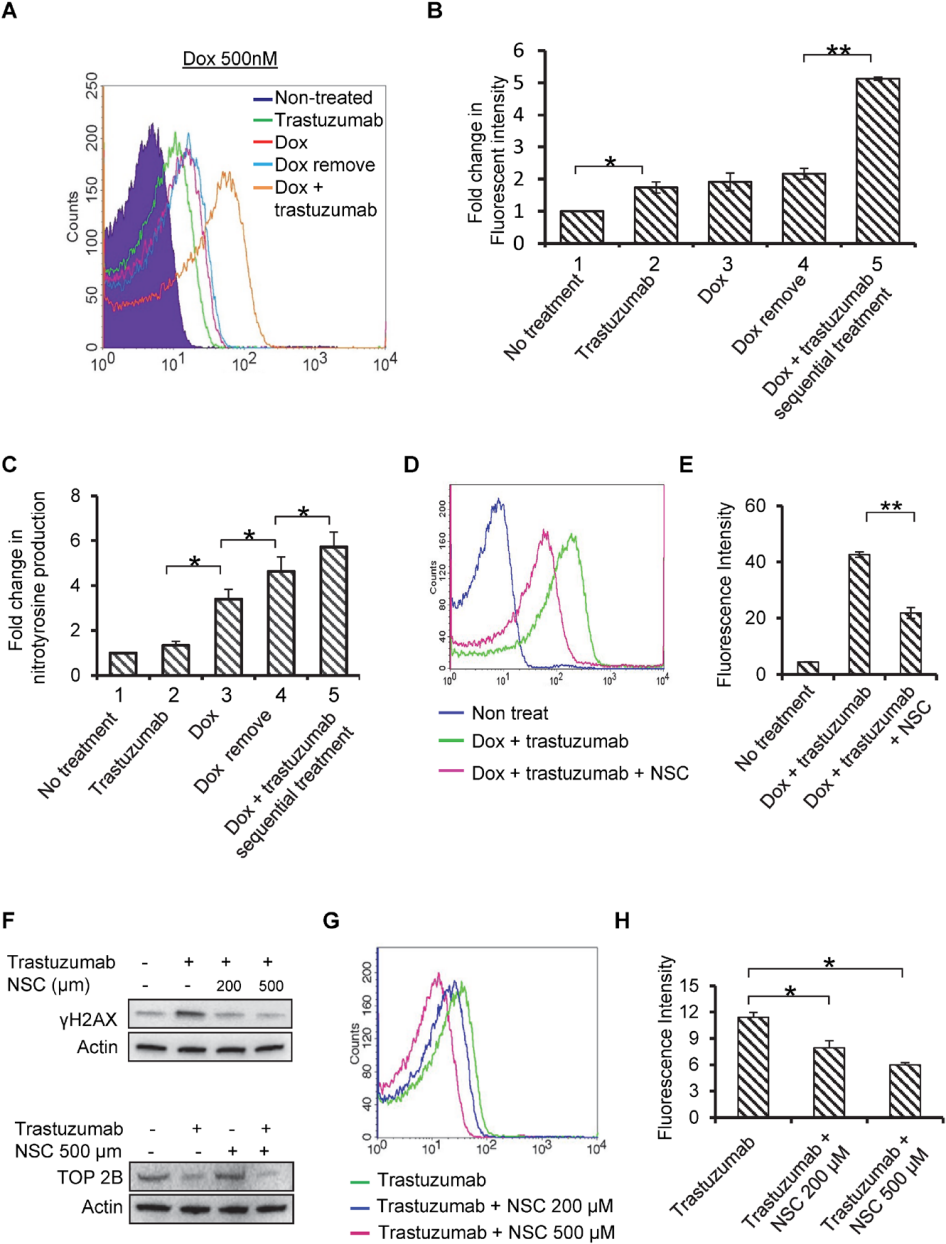
Trastuzumab enhances Dox-induced ROS and RNS production, and Rac1 inhibitor prevents trastuzumab from inducing H2AX phosphorylation and ROS production (**A**) The experiment design was essentially the same as that described in Figure 5A, except that the harvested cells were subjected to flow cytometer. (**B**) Quantitative analysis of the fold changes in fluorescence intensity obtained from flow cytometry was presented in the form of bar diagram. (**C**) Human cardiomyocytes were treated as described in Figure 5A. After treatments, cells were harvested and levels of nitrotyrosine were detected using OxiSelectTM Nitrotyrosine ELISA kit according to manufacturer’s protocol, and data was presented in the form of bar diagram. (**D**) Human cardiomyocytes were treated with Dox (0.5 µM) + trastuzumab (50 µg/ml), Dox (0.5 µM) + trastuzumab (50 µg/ml) + NSC (500 µM) or left untreated for 24h, and ROS production was detected by DCFDA staining. (**E**) Quantitative analysis of fluorescence intensity obtained in Figure 6D was presented in the form of bar diagram. (**F**) Human primary cardiomyocytes were treated with trastuzumab alone or in combination with NSC23766 (NSC) at 200 µM and 500 µM for 48h. After treatment, Western blotting analysis was performed to detect the phosphorylation levels of H2AX using anti-γH2AX antibody (Upper panel). TOP2 B expression was detected in human cardiomyocytes after 24 h treatment with trastuzumab (50 µg/ml), NSC (500 µM) or combination (Lower panel). (**G**) Human primary cardiomyocytes were treated as described in Figure 6F, and ROS production was measured as described in Figure 6A. (**H**) Quantitative analysis of fluorescence intensity obtained from flow cytometry in Figure 6G was presented in the form of bar diagram. Data in Figure 6 are representative of two or more independent experiments and expressed as mean ± SEM. Statistical significance was determined by the Student’s *t*-test; ^*^P < 0.05; ^**^P < 0.01.

Enhanced oxidative stress has been shown to play significant role in pathogenesis of cardiac abnormalities. In our earlier study, we have demonstrated that trastuzumab increases the levels of biomarkers of oxidative stress (3-nitrotyrosine and 4-hydroxynonenal) in heart tissues of trastuzumab-treated mice [21]. We have also demonstrated that trastuzumab-mediated autophagy inhibition leads to significant increase in generation of ROS in human cardiomyocytes [23]. In this study, we assessed whether sequential treatment of doxorubicin followed by trastuzumab enhances oxidative stress in cardiomyocytes compared with doxorubicin alone. Cardiomyocytes were treated as described in Figure 5A. After treatment, the cells were subjected to DCFDA staining to monitor the ROS production. Figure 6A and 6B showed that treatment of doxorubicin followed by trastuzumab significantly enhances ROS generation compared with doxorubicin alone (Figure 6A and 6B).

3-nitrotyrosine (3NT) has been widely utilized as a marker for the production of reactive nitrogen species such as (ONOO−, ·NO2, etc). Experimental design for Figure 6C is essentially the same as that showed in Figure 6A, except that the cells were measured for the 3NT production. As shown in Figure 6C, 3NT production was significantly increased in sequential treatment of doxorubicin followed by trastuzumab as compared with that treated with doxorubicin only. These data suggest that doxorubicin therapy augmented the nitration of tyrosine residues and nitrative stress conditions in human cardiomyocytes, which were further amplified by sequential treatment with trastuzumab (Figure 6C).

### Rac1 inhibitor, NSC23766, rescues ROS production and DNA damage induced by trastuzumab alone and trastuzumab + doxorubicin

Rac1, a Ras-like small GTPase, contributes to doxorubicin-mediated apoptosis and oxidative stress in cardiomyocyte [17]. Rac1-specific inhibitor NSC23766 (NSC) has been shown to exhibit protective effects in mitigating doxorubicin-induced cardiotoxicity by reducing doxorubicin-induced γH2AX levels and repairing DNA damage response [18]. We investigated whether NSC was able to rescue cardiomyocytes from trastuzumab or trastuzumab + doxorubicin induced ROS production and DNA damage. Combining NSC with doxorubicin + trastuzumab significantly decreased ROS levels in cardiomyocytes, as detected by DCFDA flow cytometry staining (Figure 6D and 6E). In addition, Figure 6F showed that NSC prevented trastuzumab from increasing γH2AX levels in cardiomyocytes, indicating that Rac1 activity is involved in trastuzumab-induced TOP2B inhibition. Moreover, NSC itself had minimal impact on TOP2B expression level (Figure 6F). Trastuzumab-induced ROS generation was also mitigated by NSC intervention, suggesting that inhibition of Rac1 signaling can effectively antagonize cardiotoxicity induced by either trastuzumab alone and doxorubicin + trastuzumab combination (Figure 6G and 6H). Taken together, these data further support the idea that by targeting TOP2B, trastuzumab induces DNA damage response and ROS production. Given the involvement of Rac1 in cardiac DNA damage response and ROS production induced by doxorubicin and trastuzumab, dual targeting of TOP2B by doxorubicin and trastuzumab would lead to a synergistic toxicity on cardiomyocytes.

## DISCUSSION

Despite combination therapy of anthracycline and trastuzumab significantly increases the cardiotoxicity in cancer patients; little research effort has been invested in exploring the molecular mechanisms of cardiotoxicity induced by these dual agents. A repair-interfering model was previously proposed regarding the mechanisms induced by the combination of anthracycline and trastuzumab [24]. Specifically, anthracyclines damage cardiac myocytes via mechanisms that disrupt the TOP2 function, leading to oxidative stress or cell death. In the absence of trastuzumab, the damaged cells may undergo cell repairing. However, this repairing process could be interfered by trastuzumab, resulting in a greater proportion of myocytes undergoing cell death. This model was proposed based on little experimental evidence available at the molecular level as to how trastuzumab induces cardiotoxicity. Doxorubicin and trastuzumab are two different types of agents, but they both can cause ROS stress and subsequent damage in human cardiomyocyte. It is tempting to speculate that these two drugs may exert their effects through a closely related mechanism. We recently reported that trastuzumab dysregulates HER2 signaling to mediate increase in ROS production in human primary cardiomyocytes [23]. In the present study, we provide a novel molecular connection between trastuzumab and anthracycline, which further advances our understanding of molecular mechanisms of cardiotoxicity induced by the combination of anthracyclines and trastuzumab. Based on data presented in this study and literature [24], both anthracyclines and trastuzumab can downregulate TOP2B in cardiomyocytes, leading to the enhanced double-strand DNA damage and the increase in ROS production. Because TOP2B has been previously discovered as a major target responsible for the cardiotoxicity induced by doxorubicin [24], it is intuitive that trastuzumab enhances doxorubicin’s toxic effect to heart by rendering further inhibition of TOP2B in cardiomyocytes via either concurrent or sequential treatment. In the present study, we provide evidence demonstrating that either concurrent or sequential treatment of cardiomyocytes with anthracyclines and trastuzumab significantly reduces TOP2B protein levels, increases apoptotic activity and ROS production, and inhibits the cardiomyocyte growth. Furthermore, we find that doxorubicin treatment enhances the levels of HER2 expression and upregulates downstream of HER2 signaling in cardiomyocytes. While this could be either a survival or compensatory machinery of cardiomyocytes under the stressed condition induced by doxorubicin, it may render the cardiomyocytes to become addictive to HER2 signaling for survival under stressed condition. On the other hand, it may also leave cardiomyocytes to become more sensitive to trastuzumab treatment than that without HER2 upregulation. Our findings provide molecular basis for the enhanced cardiotoxicity induced by the combination of anthracyclines and trastuzumab. We propose an additive or synergistic model of cardiotoxicity induced by combination of anthracyclines and trastuzumab although we believe that further impacts of trastuzumab treatment on cardiac myocytes can interfere the recovery of cardiac myocytes that have been injured by anthracyclines. Our study suggests that TOP2B is a valuable candidate that can be used to mitigate the cardiotoxicity induced by this combination therapy for HER2-positive breast cancer patients.

## MATERIALS AND METHODS

### Antibodies and reagents

Antibodies against TOP2B and γH2AX were obtained from Bethyl Laboratories. Antibodies against actin and phosphorylated ATR (S428) were obtained from Sigma-Aldrich. Antibody against TOP2A was obtained from Origene. The secondary antibodies used for Western blotting and fluorochrome-conjugated secondary antibodies used for immunofluorescence studies were purchased from Invitrogen/Molecular Probes. Pharmaceutical grade trastuzumab (Genentech), cetuximab (ImClone LLC), ramucirumab (Eli Lilly and Company), pertuzumab (Genentech) and doxorubicin were purchased from the pharmacy at the National Institutes of Health (NIH). ROS detection reagent 2′-7′-dichlorodihydrofluorescein diacetate (DCFDA) was purchased from Life Technologies. The OxiSelect™ Nitrotyrosine ELISA Kit was procured from Cell BioLabs.

### Cell culture and treatments

Human primary cardiomyocytes were obtained from ScienCell research laboratories (cat#6200). Cells were cultured in human cardiac myocytes media (ScienCell) containing 5% FBS, and 1% penicillin/streptomycin solution and 1% cardiac myocytes growth supplement (ScienCell) and used within 6 months. These primary cell lines were not authenticated by the authors. For trastuzumab and pertuzumab treatment, cardiomyocytes were seeded in 6-well plates in 2-ml pre-warmed growth media. The concentration of trastuzumab was used at 50 μg/ml or left untreated. Concentrations of doxorubicin and treatment conditions were described in figure legends for each experiment.

### Microarray experiment and analysis

Human cardiomyocytes were treated with trastuzumab (50 µg/ml), trastuzumab (50 µg/ml) plus pertuzumab (50 µg/ml) or T-DM1 (10 µg/ml) for 24h. After incubation, cells were harvested and total RNA was extracted using Trizol RNA isolation reagent (Thermofisher Scientific) according to manufacturer’s instructions. Microarray experiment and analysis were performed at National Institute for Diabetes and Digestive and Kidney Diseases (NIDDK, Bethesda, MD) Genomic core facility. Total RNA at the concentration of approximately 200–800 ng/µl of each treatment group in three replicates was provided to NIDDK Genomic core facility. The quality of RNA was determined using bioanalyzer before processing the samples. GeneChip Human Gene 2.0 ST Array (catalog # 902112 Affymetrix) was used to hybridize the cDNA.

### TUNEL assay

TUNEL assay was carried out with DeadEnd™ Fluorometric TUNEL System according to manufacturer’s instruction (Promega). Briefly, cells were grown on coverslip, treated with drugs and fixed in 4% formaldehyde at RT for 20 min, followed by permeablization with 0.2% Triton^®^ X-100 in PBS for 5 min. Cells were then rinsed twice in PBS and equilibrated with TdT enzymatic buffer provided in the kit. 50 µl of terminal transferase and fluorescently labeled nucleotide mix with buffer was used for each assay, and the enzymatic reaction was allowed to proceed for 60 min at 37°C. Negative control was setup as the reaction solution without terminal transferase. After reaction, cells were rinsed with PBS and then washed with 2x SSC for 15 min. Lastly, cells were rinsed twice with PBS and mounted with DAPI reagent for counterstaining cell nucleus. Fluorescent images were acquired with ZEISS confocal microscope model LSM880.

### Nitrotyrosine assay

The OxiSelect™ Nitrotyrosine ELISA Kit is a competitive enzyme immunoassay which is used for rapid detection and quantitation of 3-nitrotyrosine in protein sample. Human cardiomyocytes were treated as indicated in figure legend and assay was performed according to manufacturer’s instructions (Cell BioLabs Inc). The quantity of 3-nitrotyrosine in protein sample is determined by comparing its absorbance with that of a known nitrated BSA standard curve. Each sample was assayed in triplicate, and absorbance was read on spectrophotometer using 450 nm wavelength. The protein nitrotyrosine content in treatment groups was determined by comparing with a standard curve that is prepared from predetermined nitrated BSA standards and presented in the form of fold change in NT production.

### ROS detection by flow cytometry

Cell permeable reagent 2’,7’-dichlorofluorescin diacetate (DCFDA) is a fluorogenic dye that measures reactive oxygen intermediates in cells and has been widely used as an indicator for oxidative stress. After diffusion into the cell, DCFDA is transformed into highly fluorescent compound 2’,7’-dichlorofluorescein (DCF), which can be detected by flow cytometry with maximum excitation and emission spectra of 488nm and 535 nm respectively. Briefly, human cardiomyocytes were treated with doxorubicin, trastuzumab or combination of two drugs and then harvested, washed with PBS and re-suspended in PBS. Cells were stained with DCFDA at 10 µM concentration and incubated for 30 min at 37°C. After staining, cells were washed, suspended in PBS and analyzed by flow cytometer with excitation at 488 nm and emission at 530 nm wavelength. Experiment was performed in triplicate and the fold changes in fluorescent intensity between non-treated and treated groups were quantified and presented in the form of bar graph.

### Caspase 3/7 assay

Primary human cardiomyocytes were grown in 96-well plates and treated with trastuzumab (50 µg/ml) and doxorubicin (500 nM) accordingly. After treatment, cells were washed once with 1x PBS, and 100 µl PBS mixed with 100 µl reconstituted caspase 3/7 substrate from Apo-ONE^®^ Homogeneous Caspase-3/7 Assay kit (Promega) were added to each well of the 96-well plates. Solutions were gently mixed. The assay was preceded for 1h at room temperature. Blank and negative controls were used to identify the positive signal. When the reaction was finished, fluorescent signal from the cleaved substrate was recorded with FilterMax F3 plate reader equipped with Rhodamine 110 filter set (Molecular Devices Inc.), and cell numbers were measured with Celigo imaging system.

### Immunofluorescent confocal microscopy imaging

5 × 10^4^ cells were seeded on a cover glass (18 mm) in a well of 12-well plate, and cells were grown and treated for the experiment. After treatment, cells were rinsed with PBS for twice and fixed with 1 ml of 4% formaldehyde for 15 min at room temperature. Next, cells were rinsed with PBS again and permeabilized with 1ml 0.2% Triton X-100 in PBS for 10 min, followed by block with 10% donkey serum in PBS for 1h. The primary antibody diluted at 1:100 with staining buffer (5% Donkey serum in PBS with 0.2% Triton X-100) was added to the coverslip and incubated at 4°C overnight. Subsequently, the coverslip was washed with staining buffer twice, 5 min each time. Incubation with secondary antibody conjugated with fluorescent dye was done at 1:500 dilutions at room temperature for 2h in staining buffer. In the end, the cover slip was washed 3 times, 5 min each and mounted on a glass slide using ProLong Gold antifade reagent with DAPI (Life Technologies). ZEISS confocal microscope LSM880 was used to acquire the images, and fluorescent signal intensity and quantitative analysis was conducted with ImageJ software (National Institutes of Health).

### Statistical analysis

GraphPad Prism and Microsoft Excel software were used for statistical studies. Statistical significance was determined by the Student's *t*-test (^*^P < 0.05; ^**^P < 0.01; ^***^P < 0.001). Data are expressed as mean ± SEM.
